# Astaxanthin uptake in domestic dogs and cats

**DOI:** 10.1186/1743-7075-7-52

**Published:** 2010-06-21

**Authors:** Jean Soon Park, Hong Wook Kim, Bridget D Mathison, Michael G Hayek, Stefan Massimino, Gregory A Reinhart, Boon P Chew

**Affiliations:** 1 School of Food Science, Washington State University, Pullman, WA 99164-6376, USA; 2 P&G Pet Care, Lewisburg, OH 45338, USA

## Abstract

**Background:**

Research on the uptake and transport of astaxanthin is lacking in most species. We studied the uptake of astaxanthin by plasma, lipoproteins and leukocytes in domestic dogs and cats.

**Methods:**

Mature female Beagle dogs (18 to 19 mo old; 11 to 14 kg BW) were dosed orally with 0, 0.1, 0.5, 2.5, 10 or 40 mg astaxanthin and blood taken at 0, 3, 6, 9, 12, 18 and 24 h post-administration (n = 8/treatment). Similarly, mature domestic short hair cats (12 mo old; 3 to 3.5 kg body weight) were fed a single dose of 0, 0.02, 0.08, 0.4, 2, 5, or 10 mg astaxanthin and blood taken (n = 8/treatment) at the same interval.

**Results:**

Both dogs and cats showed similar biokinetic profiles. Maximal astaxanthin concentration in plasma was approximately 0.14 μmol/L in both species, and was observed at 6 h post-dosing. The plasma astaxanthin elimination half-life was 9 to 18 h. Astaxanthin was still detectable by 24 h in both species. In a subsequent study, dogs and cats were fed similar doses of astaxanthin daily for 15 to 16 d and astaxanthin uptake by plasma, lipoproteins, and leukocytes studied. In both species, plasma astaxanthin concentrations generally continued to increase through d 15 or 16 of supplementation. The astaxanthin was mainly associated with high density lipoprotein (HDL). In blood leukocytes, approximately half of the total astaxanthin was found in the mitochondria, with significant amounts also associated with the microsomes and nuclei.

**Conclusion:**

Dogs and cats absorb astaxanthin from the diet. In the blood, the astaxanthin is mainly associated with HDL, and is taken up by blood leukocytes, where it is distributed to all subcellular organelles. Certain aspects of the biokinetic uptake of astaxanthin in dogs and cats are similar to that in humans.

## Introduction

Research has shown that carotenoids play important roles in modulating immunity [1], reproduction [2], cancer [3], age-related macular degeneration, and atherosclerosis [4]. However, these studies have focused mainly on β-carotene, lutein and lycopene. Recent studies have similarly shown that astaxanthin, a ketocarotenoid, possesses important biological actions [1]. The antioxidant activity of astaxanthin has been reported to be higher than that of α-carotene, β-carotene and lutein [5,6] and α-tocopherol [7]. Astaxanthin reduced oil-induced oxidative stress [8] and lowered serum lipid peroxides and transaminase activities [9] in fish. Both in vitro and in vivo studies have shown that astaxanthin can enhance humoral [10] and cell-mediated [11] immune responses, and inhibit cancer [12,13], and suppress bacterial infection [14]. In spite of these known functions of astaxanthin, little is known concerning its uptake in most species. Our immediate objective is to study the biokinetic uptake of astaxanthin by blood, lipoproteins and leukocytes in dogs and cats; our long-term objective is to study the action of dietary astaxanthin in modulating immune health in these species.

## Materials and Methods

The comparative uptake of dietary astaxanthin in domestic dogs and cats was studied. All studies were approved by the Washington State University Institutional Animal Care and Use Committee.

### Dog study

Female Beagle dogs (18 to 19 mo old, 11 to 14 kg BW) were fed a nutritionally-balanced diet (200 g/dog/d, The Iams Co., Lewisburg, OH). The diet composition was as follows (g/kg): 66.2 moisture, 262 protein, 74.5 ash, 160 fat, 14.8 Ca, 10.3 P, and 437.3 nitrogen-free extract. All dogs were housed in 2 × 2 m pens (2 dogs/pen) in a temperature- (20 to 22°C) and light- (14 h light) controlled facility. In experiment 1, dogs were given one oral dose of 0, 0.1, 0.5, 2.5, 10 or 40 mg astaxanthin (Carophyll pink, 8% beadlet, Hoffmann La-Roche, Paramus, NJ); astaxanthin was suspended in 5 ml of water and fed orally using a feeding syringe. A preliminary study with 3 dogs was used to determine sampling times. Blood was taken from the jugular vein at 0, 3, 6, 9, 12, 18 and 24 h post-administration (n = 8/treatment).

In experiment 2, astaxanthin uptake from repeated oral doses was studied. Dogs (n = 8/treatment) were dosed once daily at 0800 h for 16 consecutive days with 0, 0.1, 0.5, 2.5, 10 or 40 mg astaxanthin. On days 0, 1, 2, 4, 6, 8, 10, 13 and 16, blood was taken 6 h after dosing; this sampling time was chosen because peak concentrations of astaxanthin were observed 6 h post-dosing in experiment 1. Plasma was collected following centrifugation at 400 × g, and analyzed by high performance liquid chromatography (HPLC). The resultant buffy coat interface following centrifugation was collected and subjected to Percoll (Histopaque-1077, Sigma-Aldrich, St. Louis, MO) centrifugation [15], and lymphocyte number determined (Coulter Electronics, Hialeah, FL). Cells were resuspended in PBS containing 30 g/L sodium ascorbate (Sigma-Aldrich) as an antioxidant, and disrupted by sonication (30 s). An aliquot of the lymphocyte homogenate was extracted for HPLC analysis of astaxanthin in whole lymphocytes. The remaining lymphocyte homogenate was subjected to subcellular fractionation as previously described [15]. Briefly, the homogenates were centrifuged to obtain the nuclear (600 × g for 10 min at 4°C), mitochondrial (17,300 × g for 20 min at 4°C), microsomal (102,000 × g for 60 min at 4°C) and cytosolic fractions. All samples were stored at -80°C prior to HPLC analysis.

#### Lipoprotein Separation and Cholesterol Analysis

Serum obtained from dogs fed 0, 2 or 10 mg astaxanthin was used to isolate lipoproteins by density gradient ultracentrifugation [15,16]. Briefly, solid KBr (0.114 g) and sucrose (0.025 g), serum (1 mL) and Sudan black (2.19 mol/L in ethylene glycol; Sigma-Aldrich) were placed in a polycarbonate centrifuge tube and subsequently overlayed with 2.4 mL salt solution (0.195 mol/L NaCl, 0.638 mol/L KBr, and 0.2 mmol/L disodium EDTA; density 1.06 g/mL), and then with 2.4 mL of 0.2 mmol/L disodium EDTA in distilled water. The mixture was then centrifuged at 232,000 × g for 7 h at 20°C (Sorvall T-865.1 fixed angle rotor, Sorvall OTD65B). The lipoprotein fractions were removed and used for HPLC analysis of astaxanthin and for cholesterol determination [15].

#### Cholesterol Analysis

Cholesterol reagent was prepared by first dissolving 520 mg ferric perchlorate in 600 mL ethyl acetate, cooled and then adding 400 mL cold concentrated sulfuric acid. The cholesterol reagent (25 mL) was added to 25 μL of sample or standard (2 mg cholesterol/L in glacial acetic acid), reacted for 1.5 min in a 100°C water bath before being cooled to <20°C. Optical density was read at 600 nm.

### Cat study

Mature female domestic short hair cats (12 mo of age; 3 to 3.5 kg body weight; Liberty Research, Waverly, NY) were fed a basal diet (The Iams Co.) containing (g/kg): 72 moisture, 310 protein, 52 ash, 217 fat, 14 crude fiber, 11.5 Ca, 8.3 P. Cats were group-housed indoors in light- (14 h light, 10 h dark) and temperature-controlled (20 - 22°C) rooms. Two to three cats were housed in each 2 × 2 m pen and had free access to food and water. In experiment 3, cats (n = 8/treatment) were given a single oral dose of 0, 0.02, 0.08, 0.4, 2, 5, or 10 mg astaxanthin; the astaxanthin was suspended in 1 mL of water and fed with a syringe. Sampling times were chosen based on a preliminary study using three cats from which blood was sampled at close intervals; blood was sampled from the jugular vein at 0, 3, 6, 9, 12, 18 and 24 h after dosing. The same oral doses of astaxanthin as above were administered daily at 0800 h for 15 consecutive days (n = 8/treatment; experiment 4). Blood was sampled once daily 6 h after each feeding based on experiment 3 peak blood astaxanthin concentrations. Plasma was separated for HPLC analysis. In addition, a larger volume of blood was taken on d 0, 6 and 15 for studying astaxanthin uptake by blood leukocytes. Due to the inconsistency in obtaining a reasonably pure population of lymphocytes, total blood leukocytes were used for subcellular fractionation as described earlier. On d 6 and 15, serum obtained from cats fed 0, 2 or 10 mg astaxanthin was used to isolate lipoproteins fractions and cholesterol as described earlier.

#### HPLC Analysis

In both studies, plasma, lymphocyte homogenate and lymphocyte subcellular fractions were analyzed for astaxanthin content by HPLC (Alliance 2690 Waters HPLC system fitted with a photodiode array detector, Waters, Milford, MA) as previously described [17]. Protein in the samples were first precipitated with an equal volume of absolute ethanol containing BHT (1 g/L) before being extracted using at least 6 volumes of a 1:1 mixture (v/v) of petroleum ether:anhydrous diethyl ether. Extraction was repeated until the pellet was clear and the solvent pooled and dried under nitrogen gas. Trans-β-apo-8'carotenal (Sigma Chem. Co., St. Louis, MO) was used as the internal standard. The mobile phase used was a mixture of acetonitrile:methanol:water (47:47:16, v/v/v), and samples were eluted through a 5-μm spherical C-18 column (3.9 × 150 mm Resolve, Waters, Milford, MA) with a flow rate of 1.5 mL/min. Absorbance was monitored at 492 nm on a photodiode array detector.

#### Statistical Analysis

Data were analyzed by repeated sampling analysis of variance using the General Linear Models of SAS. The statistical model was Yijk = μ + Treatmenti + Animalj(Treatmenti) (error A used to test the effects of treatment) + Sampling periodk + Treatmenti*Periodk + eijk (error B). Differences among treatment means within a sampling period were compared by a protected LSD test.

## Results

### Uptake in Dogs

**Figure 1:**
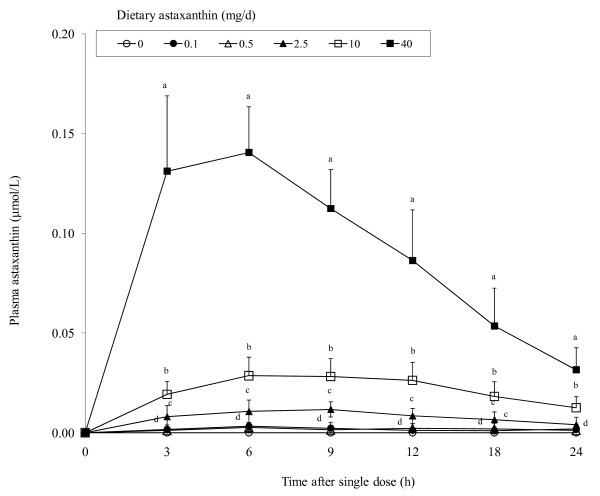
**Concentrations of plasma astaxanthin in dogs (body weight = 11 to 14 kg) given a single oral dose of 0, 0.1, 0.5, 2.5, 10 or 40 mg astaxanthin**. Values are means ± SEM (n = 8) as analyzed by repeated measures ANOVA. Means with different superscripts within a sampling period are significantly different (*P < 0.05*).

Astaxanthin was undetectable in the plasma of all dogs prior to astaxanthin supplementation, and also in unsupplemented dogs during the entire study period (Figure 1). In contrast, plasma astaxanthin increased (P < 0.01) rapidly in a dose-dependent manner by 3 h post-dosing. Plasma astaxanthin was low (0.002 μmol/L average) in dogs fed 0.1 or 0.5 mg astaxanthin. Peak plasma concentrations were generally observed around 6 h after a single oral dose. Dogs fed 40 mg astaxanthin had peak plasma astaxanthin concentrations that were 4 and 11 times higher than those fed 10 or 2.5 mg astaxanthin, respectively, indicating a disproportionately greater increase in plasma astaxanthin concentration in dogs fed 40 mg astaxanthin. Plasma astaxanthin generally started to decrease by 9 h post-dosing. However, at 24 h after dosing, astaxanthin concentrations were still higher in dogs fed 2.5, 10 or 40 mg astaxanthin when compared to control dogs. Plasma astaxanthin concentrations decreased (P < 0.01) rapidly in dogs fed 40 mg astaxanthin but much more gradually in those fed lower doses. The half-life of astaxanthin in the plasma was approximately 9 h (40 mg) to 18 h (0.5 to 10 mg groups). Astaxanthin feeding did not influence plasma α-tocopherol and retinol concentrations which averaged 9.3 ± 1.8 and 5.0 ± 0.4 μmol/L, respectively.

**Figure 2:**
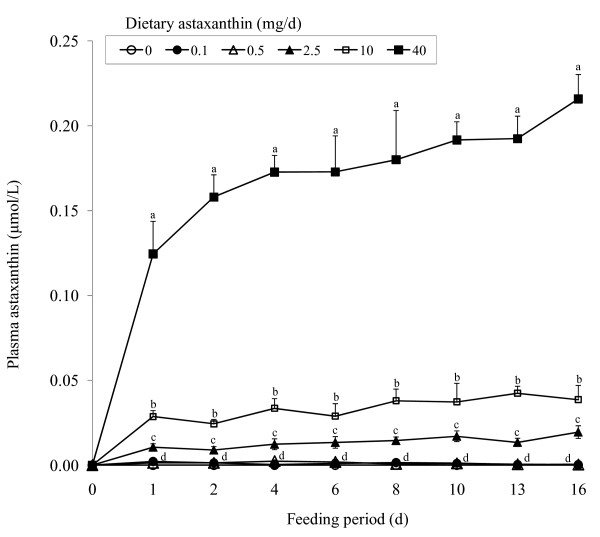
**Concentrations of plasma astaxanthin in dogs (body weight = 11 to 14 kg) administered daily doses of 0, 0.1, 0.5, 2.5, 10 or 40 mg astaxanthin orally**. Values are means ± SEM (n = 8) as analyzed by repeated measures ANOVA. Means with different superscripts within a sampling period are significantly different (*P < 0.05*).

When dogs were given daily doses of astaxanthin, plasma concentrations of astaxanthin increased (P < 0.01) dose-dependently (Figure 2). Concentrations increased (P < 0.01) rapidly after the first dose and continued to increase through d 16. In dogs fed 40 mg astaxanthin, plasma astaxanthin averaged 4 and 9 times higher (P < 0.05) compared to those fed 10 and 2.5 mg astaxanthin. Again, dietary astaxanthin did not influence plasma α-tocopherol and retinol concentrations and averaged 8.9 ± 0.6 and 5.0 ± 0.3 μmol/L, respectively.

Astaxanthin in the blood was mainly associated with the HDL fraction, with no detectable amounts in the LDL and VLDL fractions. Uptake of astaxanthin by HDL was dose-dependent (P < 0.05) and was maximal by d 6 in dogs fed 10 or 40 mg astaxanthin (averaged 2.2 ± 0.4 and 3.2 ± 0.3 nmol/mg cholesterol, or 57.2 ± 3.9 and 97.9 ± 11.8 nmol/L plasma, respectively). Astaxanthin was not detectable in any lipoprotein fraction in unsupplemented dogs.

Astaxanthin was not detectable in whole lymphocyte homogenates in control dogs and those fed 0.1 or 0.5 mg astaxanthin for up to 16 d (Table 1). However, astaxanthin was measurable in whole lymphocytes on d 4, 8 or 16 in dogs fed 40, 10 or 2.5 mg astaxanthin, respectively, and astaxanthin uptake was dose-related. Astaxanthin continued to be accumulated by circulating lymphocytes through d 16 of supplementation.

**Table 1:** Uptake and relative distribution percentages of astaxanthin in whole leukocytes and subcellular fractions in dogs fed 0, 0.1, 0.5, 2.5, 10 or 40 mg astaxanthin daily for 15d

	Treatment (mg astaxanthin/dog/d)					
	
	0	0.1	0.5	2.5	10	40
**Day 4**						
Whole leukocyte (nmol astaxanthin/10^8 ^cells)						
	nd	nd	nd	nd	nd	6.8 ± 1.7*
Subcellular fraction (% of whole leukocyte)						
Nuclei	nd	nd	nd	nd	nd	49
Mitochondria	nd	nd	nd	nd	nd	nd
Microsome	nd	nd	nd	nd	nd	51
Cytosol	nd	nd	nd	nd	nd	nd
**Day 8**						
Whole leukocyte (nmol astaxanthin/10^8 ^cells)						
	nd	nd	nd	nd	7.1 ± 1.6*	50.6 ± 11.7*
Subcellular fraction (% of whole leukocyte)						
Nuclei	nd	nd	nd	nd	49	18
Mitochondria	nd	nd	nd	nd	nd	50
Microsome	nd	nd	nd	nd	51	26
Cytosol	nd	nd	nd	nd	nd	6
**Day 16**						
Whole leukocyte (nmol astaxanthin/10^8 ^cells)						
	nd	nd	nd	2.5 ± 0.6*	21.8 ± 5.1*	66.4 ± 15.4*
Subcellular fraction (% of whole leukocyte)						
Nuclei	nd	nd	nd	45	16	22
Mitochondria	nd	nd	nd	nd	35	43
Microsome	nd	nd	nd	55	30	28
Cytosol	nd	nd	nd	nd	19	7

Total leukocyte values are means ± SEM (n = 8) as analyzed by repeated measures ANOVA.

*Means within a row are significantly different from unsupplemented animals (*P < 0.05*).

Fractionation of the peripheral blood lymphocytes revealed that the initial subcellular uptake of astaxanthin was by microsomes and nuclei, with approximately equal distribution between these two fractions (Table 1). Again, astaxanthin was measurable starting on d 4, 8 and 16 in dogs fed 40, 10 or 2.5 mg astaxanthin, respectively. By the next sampling period (d 8 for 40 mg group or d 16 for the 10 mg group), astaxanthin uptake was highest in the mitochondria (35 to 50% of total astaxanthin in lymphocytes), followed by the microsomes (26 to 30% of total astaxanthin taken up). During this latter period, the nuclei still contained a significant portion (16 to 18%) of total astaxanthin in the lymphocytes.

### Uptake in Cats

**Figure 3:**
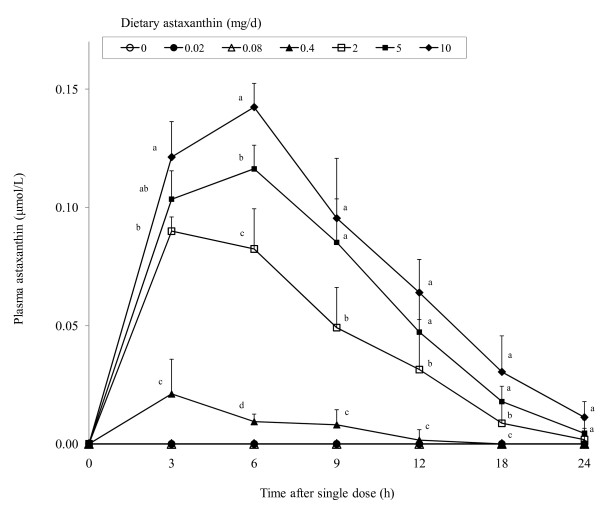
**Concentrations of plasma astaxanthin in cats (body weight = 3.0 to 3.5 kg) given a single oral dose of 0, 0.02, 0.08, 0.4, 2, 5, or 10 mg astaxanthin**. Values are means ± SEM (n = 8) as analyzed by repeated measures ANOVA. Means with different superscripts within a sampling period are significantly different (*P < 0.05*).

Uptake of dietary astaxanthin in cat plasma after a single dose was dose-dependent (Figure 3). As with dogs, astaxanthin was not detectable in unsupplemented cats. Peak plasma concentrations were generally observed between 3 to 6 h. Whereas plasma astaxanthin concentrations in cats fed 2 to 10 mg astaxanthin generally reflected dietary doses, concentrations in cats fed 0.4 mg astaxanthin or less were much lower. In fact, astaxanthin was not detectable in the plasma of cats fed 0.02 or 0.08 mg astaxanthin. At 24 h after dosing, plasma astaxanthin was still higher in cats fed 5 or 10 mg astaxanthin when compared to control cats. The plasma astaxanthin elimination half-life was approximately 6 h.

Concentrations of plasma α-tocopherol and retinol were not influenced by astaxanthin supplementation and averaged 10.4 ± 0.7 and 0.61 ± 0.04 μmol/L, respectively.

**Figure 4:**
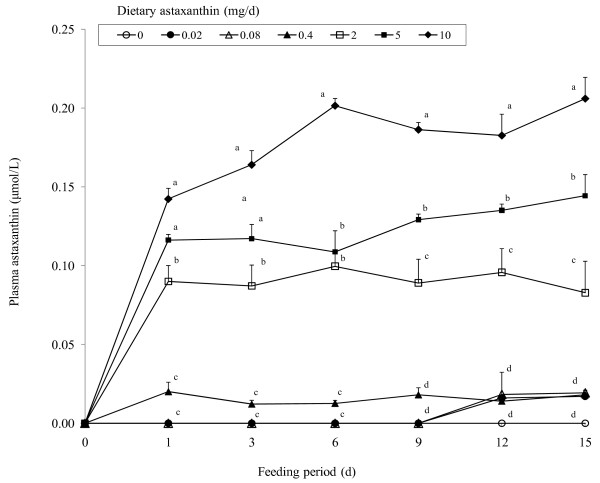
**Concentrations of plasma astaxanthin in cats (body weight = 3.0 to 3.5 kg) administered daily doses of 0, 0.02, 0.08, 0.4, 2, 5, or 10 mg astaxanthin**. Values are means ± SEM (n = 8) as analyzed by repeated measures ANOVA. Means with different superscripts within a sampling period are significantly different (*P < 0.05*).

Daily doses of astaxanthin administered for 15 d resulted in a dose-dependent increase (P < 0.01) in plasma concentrations of astaxanthin (Figure 4). Concentrations increased (P < 0.01) rapidly after the first dose and continued to increase through d 15 in cats given 5 or 10 mg astaxanthin. Plasma astaxanthin concentrations in cats given astaxanthin doses 0.08 or lower were generally undetectable. Longer term feeding of astaxanthin did not significantly alter concentrations of plasma α-tocopherol and retinol (10.5 ± 0.1 and 0.60 ± 0.01 μmol/L, respectively).

As observed in dogs, almost all astaxanthin in the blood of cats was associated with the HDL fraction. Astaxanthin incorporation into HDL was dose-dependent (P < 0.05) and was maximal by d 6 in cats fed both 2 and 10 mg astaxanthin (averaged 5.4 ± 0.2 and 18.3 ± 2.6 pmol/mg cholesterol, or 20.4 ± 0.1 and 73.4 ± 8.2 nmol/L plasma, respectively). There was no further increase in astaxanthin uptake on d 16, with astaxanthin concentrations for cats fed 2 or 10 mg averaging 3.8 ± 0.65 and 13.3 ± 0.4 pmol/mg cholesterol, or 15.6 ± 2.6 and 59.7 ± 2.6 nmol/L plasma, respectively. Astaxanthin was not detectable in any lipoprotein fraction from unsupplemented cats.

Astaxanthin uptake in whole lymphocytes of cats administered daily doses of astaxanthin increased dose-dependently as measured on d 6 and 15 of the study (Table 2). Daily dietary doses as low as 0.02 mg astaxanthin produced detectable uptake by lymphocytes on d 15.

**Table 2:** Uptake and relative distribution percentages of astaxanthin in whole leukocytes and subcellular fractions in cats fed 0, 0.02, 0.08, 0.4, 2, 5 or 10 mg astaxanthin daily for 15d

	Treatment (mg astaxanthin/cat/d)						
	
	0	0.02	0.08	0.4	2	5	10
**Day 6**							
Whole leukocyte (nmol astaxanthin/10^8 ^cells)							
	nd	nd	nd	52 ± 0*	222 ± 76*	339 ± 75*	687 ± 29*
Subcellular fraction (% of whole leukocyte)							
Nuclei	nd	nd	nd	40	40	28	23
Mitochondria	nd	nd	nd	60	41	44	50
Microsome	nd	nd	nd	nd	19	14	14
Cytosol	nd	nd	nd	nd	nd	14	13
**Day 15**							
Whole leukocyte (nmol astaxanthin/10^8 ^cells)							
	nd	44 ± 0*	49 ± 0*	84 ± 3*	169 ± 33*	357 ± 56*	468 ± 36*
Subcellular fraction (% of whole leukocyte)							
Nuclei	nd	nd	nd	36	21	24	17
Mitochondria	nd	nd	39	33	40	45	49
Microsome	nd	46	31	16	20	15	18
Cytosol	nd	54	30	16	20	16	15

Total leukocyte values are means ± SEM (n = 8) as analyzed by repeated measures ANOVA.

*Means within a row are significantly different from unsupplemented animals (*P < 0.05*).

The distribution of astaxanthin in the different subcellular lymphocyte fractions were generally dose-related (Table 2). With daily oral doses of 0.4 to 10 mg astaxanthin, there was significant uptake of astaxanthin by the nuclei and mitochondria on d 6. On d 6, astaxanthin was detectable in the microsomes and cytosol of cats fed higher doses (5 and 10 mg). However, by d 15, all astaxanthin doses produced significant uptake of astaxanthin in all subcellular fractions, including the microsomes. Regardless of dietary doses, the greatest proportion of astaxanthin seems to be associated with the mitochondria, followed by the nuclei (Table 2).

## Discussion

Interest in the biological activity of astaxanthin has increased recently due to its role as an antioxidant [5], in enhancing humoral [10] and cell-mediated [11] immune responses, in inhibiting mammary [12] and bladder [13] cancer growth, and in gene regulation [18]. As an antioxidant, astaxanthin has been shown to possess relatively higher activity as compared to α-carotene, β-carotene and lutein [6]. In fact, the antioxidant activity of astaxanthin against certain kinds of reactive oxygen under several experimental conditions has been observed to be stronger than that of α-tocopherol [7].

Aside from a study of rainbow trout [19,20], little is known concerning the uptake and metabolism of astaxanthin. In this study, we showed that both domestic dogs and cats absorbed astaxanthin when administered orally. The maximum concentration of astaxanthin in plasma of both dogs and cats after a single dose of approximately 3.3 mg astaxanthin/kg body weight was similar in the two species and averaged 0.14 μmol/L. Osterlie et al. [21] reported that the maximum plasma astaxanthin concentration in 3 human subjects given a single dose of 100 mg astaxanthin (equivalent to approximately 1.1 mg astaxanthin/kg body weight) was 2.3 μmol/L. Therefore, the efficiency of absorption of oral astaxanthin is higher in humans than in domestic dogs and cats.

Peak concentrations of astaxanthin in plasma in both species were observed 3 to 6 h postprandially in this study, which is similar to that reported in humans [21], but earlier than that reported with β-carotene in cats at 12 to 24 h [22], humans at 24 to 48 h [23,24], and preruminant calves at 12 h to 30 h [25]. However, peak plasma β-carotene in dogs also was observed at 6 h after a single dose [26].

Maximal concentrations of astaxanthin in dogs and cats after a single dose were also lower than β-carotene in cats [22], preruminant calves [25], and ferrets [27], but higher than β-carotene uptake in dogs [26]. In contrast, mice showed a 50 fold higher uptake of oral astaxanthin compared to β-carotene [22,26,28,29] when fed higher amounts of astaxanthin (0.02 to 0.4% of the diet). Therefore, large species differences occur in the rate of absorption of different carotenoids.

In this study, plasma astaxanthin concentrations continued to increase in both dogs and cats to above 0.2 μmol/L after 15 or 16 d of astaxanthin administration, even though the rate of increase was much lower after the first day. Therefore, plasma saturation concentration for astaxanthin in dogs and cats cannot be deduced from this study. We [17] recently reported similar concentrations (0.1 μmol/L) of plasma astaxanthin in 28 human subjects given 2 to 8 mg astaxanthin daily for 8 wk. In the latter study, plasma saturation was observed after 4 wk feeding. Oral astaxanthin is generally more bioavailable in cats than in dogs especially when compared across multiple doses. This may be due to a lack of the 15,15 monoxygenase (or other oxygenases) in cats, resulting in the reduced cleavage of astaxanthin in the intestine. Alternatively, the ad libitum feeding of cats may have resulted in multiple spikes in astaxanthin absorption as opposed to the consumption of two meals by the dogs.

Plasma astaxanthin elimination half-life was longer in dogs (9 to 18 h) than in cats (6 h), and was still detectable 24 h after dosing. The elimination rate may be more rapid in these species, since the half-life of 11 to 32 h reported in humans is longer [21], and may partly explain the lower plasma concentrations. The plasma β-carotene elimination rate in dogs which showed undetectable amounts 24 h after an individual dose [26] is more rapid than in cats which had detectable levels at 72 h [22], ferrets at 75 h [27], preruminant calves at 240 h [25], and humans at 120 h [30].

Carotenoids are absorbed in the intestinal mucosa and then transported in the blood in association with plasma lipoproteins [31]. Carotenoids associated with chylomicra are transported via the lymph and blood to the liver where they are partly re-secreted with lipoproteins. High density lipoprotein is the major lipoprotein in the blood of dogs and cats. In this study, astaxanthin in the blood was present predominantly in HDL in both dogs and cats. Maximal uptake by the HDL occurred by d 6 of feeding in both species and was dose-related. In humans, most of the astaxanthin was found in the VLDL chylomicra (36 to 64%), with lesser, equal amounts distributed between the LDL and HDL [21]. The distribution of polar xanthophylls in lipoproteins is different from non-polar carotenoids [32]. Whereas carotenes are mainly transported in LDL, xanthophylls tend be found more equally distributed between HDL and LDL [33,34].

Examination of astaxanthin uptake by blood leukocytes again revealed similarities between dogs and cats. The mitochondria accounted for 40 to 50% of total astaxanthin taken up by blood leukocytes in both species while the microsomes and nuclei also took up significant amounts of astaxanthin. No reports are available on the subcellular distribution of astaxanthin in blood leukocytes from other species for the purpose of comparisons. We have previously reported subcellular uptake of dietary β-carotene in these and other species. Mitochondria again accounted for 40 to 52% of total β-carotene uptake by blood leukocytes in cats [22] and calves [15], but was lower in dogs with only 14 to 17% [26]. Uptake of β-carotene by blood leukocytes and leukocyte subcellular fractions also has been reported in humans [35], and pigs [36]. The presence of astaxanthin in the various subcellular organelles suggests the involvement of this antioxidant in cellular function. The mitochondria, through their electron transport system, utilize about 85% of the total oxygen consumed by the cell, thereby producing large quantities of reactive oxygen species [37]. Indeed, changes in mitochondrial and plasma membrane potential can influence cell-mediated immune function [37]. Also, astaxanthin supplementation in vitamin E-deficient rats protected mitochondria and erythrocyte ghosts from Fe-catalyzed lipid peroxidation [38]. In vivo, astaxanthin is located in membranes that contain a large amount of polyunsaturated fatty acids [39], and is therefore strategically located to afford protection against lipid peroxidation. Previous reports have shown that astaxanthin is more effective than β-carotene in decreasing the rate of formation of methyl linoleate hydroperoxides [40] and in preventing lipid peroxidation in rat liver microsomes [41]. Astaxanthin, in nanomolar concentrations, was able to prevent paraquat-induced oxidative stress in chicken embryo fibroblasts [42]; in the latter study, the activity of astaxanthin surpassed that of α-tocopherol and β-carotene. The high antioxidant activity of astaxanthin is attributed to the presence of a keto- and OH- group in each ionone ring which allows the molecule to be esterified, and makes it more polar than other carotenoids. Xanthophylls are thought to be located in the cell membrane lipid bilayer with their hydrophobic groups in the aqueous phase or anchored in the polar head region of the lipid bilayer [43]; this increases membrane rigidity and allows them to serve as molecular rivets to impair the entry of small polar molecules such as singlet oxygen.

The stereoisomer form of astaxanthin was not identified in this study. However, there is a preferential uptake of the Z-isomers as compared to the all-E-astaxanthin in humans [21]. The source of astaxanthin in this study is *Haematococcus pluvialis*; astaxanthin exists primarily as a monoester and in the form of the 3S,3'S enantiomer. Synthetic astaxanthin [21], on the other hand, contains primarily the 3R,3'S form.

In summary, domestic dogs and cats fed astaxanthin generally showed different biokinetic profiles when compared to humans and other species. Whether astaxanthin supplementation can modulate immune and anti-inflammatory/antioxidative function remains to be elucidated. However, this study provides target dietary doses to use in functional studies in dogs and cats.

## Competing interests

The authors declare that they have no competing interests.

## Authors' contributions

JSP and BPC designed research, analyzed data, and wrote the paper; JSP, BPC, BDM, HWK conducted research; MGH, SM, GAR provided essential materials; BPC had primary responsibility for final content. All authors read and approved the final manuscript.
